# The Relationship Between Aortic Knob Width and Metabolic Syndrome in Women with Polycystic Ovary Syndrome

**DOI:** 10.3390/jcm15135273

**Published:** 2026-07-06

**Authors:** Kıymet İclal Ayaydın Yılmaz, Emre Yılmaz, Sencer Çamcı

**Affiliations:** 1 Department of Obstetrics and Gynecology, Giresun University Faculty of Medicine, 28200 Giresun, Türkiye; iclal.ayaydin@giresun.edu.tr; 2 Department of Cardiology, Giresun University Faculty of Medicine, 28200 Giresun, Türkiye; emre.yilmaz@giresun.edu.tr

**Keywords:** polycystic ovary syndrome, aortic knob width, metabolic syndrome, cardiometabolic risk, subclinical atherosclerosis

## Abstract

**Background:** Polycystic ovary syndrome (PCOS) is a prevalent endocrine disorder that elevates the risk of cardiometabolic conditions, including metabolic syndrome (MetS). Aortic knob width (AKW) is a simple radiographic marker reflecting cumulative vascular remodeling and subclinical atherosclerosis. AKW has been associated with cardiometabolic disorders in various populations. However, its clinical relevance in women with PCOS remains unclear. This study aimed to evaluate AKW in women with PCOS and investigate its relationship with MetS. **Methods:** This retrospective case–control study comprised 200 women diagnosed with PCOS in accordance with the Rotterdam criteria, alongside 200 healthy controls matched for age and body mass index. Clinical, anthropometric, biochemical, and hormonal parameters were recorded. AKW was measured on posteroanterior chest radiographs by two independent observers. MetS was defined based on the 2009 international consensus criteria. Associations between AKW and MetS components were assessed using correlation and covariance analyses. Receiver operating characteristic (ROC) curve analysis was performed to evaluate the diagnostic performance of AKW in detecting MetS. **Results:** The AKW was significantly higher in the PCOS group than in the control group (30.43 ± 5.02 mm vs. 28.47 ± 5.21 mm, *p* < 0.001). The prevalence of MetS was markedly higher in women with PCOS (39%) than in the control group (9.5%). AKW increased progressively with the number of MetS components, with a more pronounced trend in the PCOS group. Significant correlations were observed between AKW and all MetS components, including blood pressure, waist circumference, fasting glucose, and triglycerides (all *p* < 0.001), while high-density lipoprotein cholesterol showed an inverse correlation. ROC analysis demonstrated moderate-to-good discriminatory performance of AKW for identifying MetS in women with PCOS (area under the curve: 0.80; 95% confidence interval: 0.72–0.86; *p* < 0.001), with an optimal cutoff value of 33.05 mm (78% sensitivity and 80% specificity). **Conclusions:** AKW was significantly associated with the presence and burden of metabolic syndrome among women with PCOS. Although AKW demonstrated moderate-to-good discriminatory performance for identifying MetS, prospective multicenter studies with external validation are required before its potential clinical utility can be established.

## 1. Introduction

Polycystic ovary syndrome (PCOS) is a widespread reproductive-age endocrine disorder, characterized by lifelong metabolic implications and significant health risks [1]. Although there is strong evidence suggesting that complex interactions between genetic, behavioral, and environmental factors contribute to the development of PCOS, its exact etiology and pathogenesis remain unclear [2,3].

In addition to chronic anovulation and hyperandrogenism, PCOS is also associated with numerous metabolic and endocrine disorders [4,5]. A significant proportion of women with PCOS have obesity and insulin resistance [6]. It has also been found that subclinical inflammation is common in this condition and is associated with insulin resistance [7,8]. Clearly, these factors contribute to the increased prevalence of type 2 diabetes mellitus (DM) and cardiovascular disease (CVD) in women with PCOS [6,9,10]. These patients exhibit an increased prevalence of type 2 DM, glucose intolerance, and insulin resistance regardless of body mass index (BMI). They also face a significantly higher risk of dyslipidemia, subclinical atherosclerosis, and metabolic syndrome (MetS) [4,5].

MetS is characterized by the presence of metabolic abnormalities, including insulin resistance, impaired glucose metabolism, abdominal obesity, dyslipidemia, and high blood pressure. Currently, MetS affects approximately 20% of women of childbearing age [11,12]. MetS is a definition established in 2009 through a consensus report by international scientific societies, encompassing an increased waist circumference (WC), reduced high-density lipoprotein (HDL) cholesterol levels, elevated triglyceride (TG) levels, impaired fasting glucose, and hypertension, or the presence of pharmacological treatments for these conditions. To diagnose MetS, at least three of these criteria must be met [13]. Numerous studies have shown that MetS is associated with an increased risk of developing type 2 DM and CVD [14,15]. A higher risk of developing MetS has been demonstrated in patients with PCOS, a finding that has also been confirmed in non-obese women with PCOS, compared to healthy controls matched for BMI [16,17]. MetS is a condition that poses a higher risk of cardiovascular mortality and morbidity than the sum of the risks posed by its individual components and risk factors [18].

Aortic knob width (AKW) is a radiological measurement representing the widest transverse diameter of the aortic knob—formed by the aortic arch and the proximal descending thoracic aorta—as measured from the left lateral border of the trachea on a posteroanterior chest radiograph [19]. Studies have shown that an increase in AKW is associated with MetS, mortality, cardiovascular events, heart rate variability, hypertension, and subclinical left ventricular dysfunction [20,21,22,23]. AKW offers significant clinical advantages compared to traditional vascular markers. This cost-effective parameter is widely available. It can be easily detected using routine chest radiographs, which involve low radiation exposure [24]. AKW, which represents a combination of aortic stiffening, dilation, and wall thickening, reflects not only local vascular changes but also the cumulative burden of systemic atherosclerosis [25]. Its close association with measures of atherosclerosis, such as the cardio-ankle vascular stiffness index and carotid intima-media thickness (CIMT), has also been demonstrated, and it has been studied in numerous patient groups [26,27]. Compared with established vascular markers such as carotid intima-media thickness and pulse wave velocity, AKW can be obtained from routinely performed chest radiographs without additional equipment, specialized training, or procedural costs. This characteristic may increase its practicality and accessibility in routine clinical settings. However, information regarding AKW measurements in women with PCOS is limited. In this study, we aimed to evaluate AKW measurements in women with PCOS compared to age- and BMI-matched healthy women and to investigate the association between AKW and MetS in women with PCOS.

## 2. Materials and Methods

### 2.1. Study Population

This retrospective case–control study used data from patients who were followed up at the outpatient clinic of the Department of Obstetrics and Gynecology at Giresun University Faculty of Medicine. The Scientific Research Ethics Committee of Giresun Training and Research Hospital approved the study (Protocol Code: 04.02.2026/12; Date of Approval: 4 February 2026). Each stage of the study was conducted in accordance with the principles of the Declaration of Helsinki.

The study evaluated two groups: a patient group consisting of women with PCOS and a control group of healthy women who were matched by age and BMI. The PCOS patients included in the study were selected from those followed at our clinic between 1 January 2024 and 31 December 2025. Participants were required to have undergone a chest radiograph within the past three months during their initial outpatient clinic visit. The PCOS patients in our study were selected according to the Rotterdam criteria [28].

According to the Rotterdam criteria, a diagnosis requires the presence of two of the following three conditions: oligo/anovulation accompanied by oligomenorrhea, evidence of biochemical or clinical hyperandrogenism, and an ultrasonographic morphology of polycystic ovaries characterized by the presence of ≥12 follicles with a diameter of 2–8 mm in at least one ovary [28]. Based on the inclusion and exclusion criteria, 200 patients with PCOS were enrolled in the study as a result of these evaluations.

A control group of 200 healthy premenopausal women, matched for age and BMI, who presented to the outpatient clinic for general checkups or due to non-endocrine gynecological issues, was included. The clinical, biochemical, and hormonal values, as well as the ultrasound findings of the ovaries, were normal for the women in the control group. There was no history of chronic medication use or metabolic diseases. Patients presenting with clinical signs of hyperandrogenism, such as hirsutism and acne, were excluded from the control group. Control participants were selected from women attending the same institution during the study period who did not meet the Rotterdam diagnostic criteria for PCOS and who fulfilled all inclusion and exclusion criteria.

The inclusion criteria for both groups were as follows: women of reproductive age (18–40 years old), a chest radiograph taken within the three months prior to the index outpatient visit, and no history of medication use for at least three months prior to the study. Because this was a retrospective study, women who had undergone posteroanterior chest radiographs as part of routine clinical evaluations by non-gynecological specialties were eligible for inclusion in the study. Chest radiographs were not taken as part of routine PCOS evaluation or for the purposes of this study, but were obtained retrospectively from existing medical records. These chest radiographs were accessed and evaluated through the national personal health record system of Türkiye (e-Nabız).

The exclusion criteria were evaluated under three headings: variables that could negatively affect the diagnosis of PCOS, parameters that could negatively affect AKW measurements, and chronic diseases that could negatively affect the diagnosis of MetS.

Exclusions that may adversely affect the PCOS diagnosis:

- Various etiologies of hyperandrogenism or metabolic disorders (e.g., hyperprolactinemia [prolactin > 25 ng/mL], Cushing’s syndrome, anorexia-related amenorrhea, congenital adrenal hyperplasia),
- Androgen-producing cysts or malignancies,
- Pregnancy or lactation [beta-human chorionic gonadotropin (β-hCG) > 5 mU/mL],
- Previously diagnosed metabolic disorders,
- BMI < 17 kg/m^2^ or >45 kg/m^2^

Exclusions that may adversely affect AKW measurements:

- History of thoracic surgery or radiation therapy,
- Anomalies that alter the anatomy of the thoracic spine, such as kyphosis and scoliosis

Chronic conditions excluded because they may influence the diagnosis of MetS:

- History of CVD,
- History of cerebrovascular disease,
- Heart failure,
- DM, hypertension, renal failure,
- Acute or chronic infectious disease

These exclusions applied only to participants with previously established clinical diagnoses. Participants with elevated fasting glucose or elevated blood pressure measurements who had not been previously diagnosed with DM or hypertension remained eligible for inclusion and were classified according to the predefined MetS criteria.

### 2.2. Clinical and Biochemical Measurements

The demographic characteristics included age, BMI calculated using the formula weight (kg)/height (m^2^), smoking status, WC, and hip circumference (HC). WC and HC measurements were taken according to the American Heart Association guidelines [29]. WC was measured at the narrowest circumference between the ribcage and the iliac crest. HC was measured at the widest circumference at the level of the femoral trochanters. After a five-minute rest period, heart rate (beats per minute) and systolic (SBP) and diastolic (DBP) blood pressure (mmHg) were measured in the seated position using a validated automated oscillometric sphygmomanometer (OMRON^®^, Kyoto, Japan).

Hormone, glycemic, and lipid profile parameters were measured in the early morning (8:00–9:00 a.m.) after a 12-h fast during the follicular phase of the menstrual cycle (days 3–5). Hormonal parameters and biochemical markers, including fasting glucose, insulin, HDL cholesterol, TG, total cholesterol, and low-density lipoprotein (LDL) cholesterol were evaluated using photometric or electrochemiluminescence techniques on a Cobas^®^ 8000 Modular Analyzer (Roche Diagnostics, Rotkreuz, Switzerland). To determine insulin resistance, the homeostasis model assessment of insulin resistance (HOMA-IR) index was calculated by applying the standard formula: fasting insulin (μU/mL) × fasting glucose (mg/dL)/405 [30].

Levels of follicle-stimulating hormone (FSH), luteinizing hormone (LH), prolactin, androstenedione, dehydroepiandrosterone sulfate (DHEAS), total and free testosterone, sex hormone-binding globulin (SHBG), estradiol (E2), and results of liver and kidney function tests were evaluated in both groups.

### 2.3. Ultrasonographic Assessment

Transvaginal/transabdominal ultrasounds were performed using the Toshiba Aplio 300 (Toshiba Medical Systems Corporation, Tochigi, Japan), Philips Affiniti 70G (Philips Healthcare, Best, The Netherlands), and Philips ClearVue 650 (Philips Healthcare, Best, The Netherlands) devices. Imaging was performed during the early follicular phase in women with spontaneous menstruation and regardless of the cycle phase in amenorrheic patients. Polycystic ovary morphology was defined as an ovarian volume of more than 10 cm^3^ in at least one ovary and/or the presence of 12 or more follicles in at least one ovary [31]. Polycystic ovary morphology was defined according to the diagnostic criteria routinely used in our institution during the study period and was based on the Rotterdam consensus definition. To ensure consistency in this retrospective study, all participants were classified according to the original diagnostic assessments recorded at the time of clinical evaluation. Although different ultrasound platforms were used, examinations were performed by experienced gynecologists using standardized institutional protocols and the same diagnostic criteria for polycystic ovary morphology were applied throughout the study period.

### 2.4. Aortic Knob Width Measurement

Posteroanterior chest radiographs of the patients and controls were obtained from the local imaging system. Measurements were taken using the measurement scale provided by the imaging software (Akgun PACS v4.1.2.41; Akgun Software, Ankara, Türkiye). AKW was determined by measuring the widest transverse diameter of the aortic knob—formed by the aortic arch and the proximal descending thoracic aorta—starting from the left lateral border of the trachea on the posteroanterior chest radiographs [19]. All AKW measurements were performed on standard posteroanterior chest radiographs obtained according to institutional imaging protocols. Radiographs with inadequate image quality, marked patient rotation, thoracic deformity, or prior thoracic surgery were excluded.

### 2.5. Measurement Reproducibility and Observer Agreement

To assess measurement reproducibility, two independent observers (EY and SÇ) performed AKW measurements for all participants. Each observer repeated the measurements at separate time points to evaluate intraobserver reproducibility. Intraobserver reproducibility was assessed using intraclass correlation coefficients (ICCs) based on repeated measurements performed by the same observer. For patients in whom interobserver measurement differences were detected, the average of these measurements was calculated. The ICC for EY was 0.88 [95% confidence interval (CI): 0.82–0.93; *p* < 0.001], and the ICC for SÇ was 0.89 (95% CI: 0.83–0.94; *p* < 0.001). Interobserver agreement between the two observers was evaluated using an ICC derived from measurements obtained by both observers and was 0.82 (95% CI: 0.74–0.89; *p* < 0.001).

### 2.6. Statistical Analysis

Data analysis was conducted using SPSS for Windows, version 27 (SPSS Inc., Chicago, IL, USA). The Kolmogorov–Smirnov test was used to assess the distribution of continuous variables. Variables demonstrating normal distribution were expressed as mean ± standard deviation (SD) and compared using independent-sample *t*-tests. Variables that did not satisfy normality assumptions were expressed as median (interquartile range) and compared using Mann–Whitney U tests. Categorical variables were expressed as counts and percentages and compared using chi-square tests. Pearson correlation was employed to examine the link between MetS components and AKW. To facilitate comparison across study groups, the strength and direction of the associations were summarized graphically using a forest plot. To assess AKW trends according to the number of MetS components and the trend of each MetS component according to AKW quartiles, the “p for trend” value was calculated using a contrast test in an analysis of covariance (ANCOVA) after adjusting for age, BMI, and smoking status. ANCOVA models adjusted for age, BMI, and smoking status were used for group comparisons. No additional multivariable regression model was performed because several potentially relevant confounding variables were not consistently available in the retrospective dataset. Information regarding physical activity, family history of cardiovascular disease, socioeconomic characteristics, PCOS duration, and inflammatory biomarkers was not routinely recorded and therefore could not be reliably incorporated into multivariable analyses. Although insulin resistance (HOMA-IR) was available, it was not included as an adjustment variable because it represents a core pathophysiological feature of both PCOS and MetS and may lie on the causal pathway linking metabolic dysfunction to vascular remodeling. Therefore, adjustment was restricted to age, BMI, and smoking status, which were consistently available across the study population. A receiver operating characteristic (ROC) curve was used to evaluate the sensitivity and specificity of AKW in identifying MetS. Reliability analysis was performed using the ICC based on a two-way mixed-effects model with absolute agreement. Interobserver agreement was assessed using a two-way random-effects intraclass correlation coefficient. *p* < 0.05 (two-tailed) was considered statistically significant.

### 2.7. Data Visualization and Figure Generation

As the number of MetS components increased, trends in AKW were visualized using line graphs based on adjusted mean values. Mean AKW values for each MetS component category (0, 1, 2, and ≥3 components) were obtained via ANCOVA analysis adjusted for age, BMI, and smoking status. The graphical representation shows the adjusted mean values and corresponding CIs to illustrate the variability within each subgroup. The significance of trends was assessed using contrast tests within the ANCOVA framework, and *p*-values for overall group comparisons and trends were indicated directly on the figure to enhance interpretability.

To evaluate the trends of individual MetS components based on AKW scores, patients with PCOS were divided into four groups according to their AKW scores. After adjusting for age, BMI, and smoking status, adjusted mean values for WC, blood pressure parameters, TG, HDL cholesterol, and fasting glucose were estimated using covariance ANCOVA models.

Linear trends across the AKW quarters were assessed using contrast tests within an ANCOVA framework. Gradient bar charts displaying the adjusted mean values and their respective variability were employed to illustrate the outcomes of these trend analyses and clarify quarter-dependent alterations.

## 3. Results

The overall mean age of the study population was 24.18 ± 4.16 years, and the mean BMI was 27.06 ± 4.15 kg/m^2^. These variables were similar between the groups. While WC was significantly higher in the PCOS group (73.39 ± 11.77 cm vs. 71.98 ± 12.08 cm, *p* = 0.001), HC was similar between the groups. AKW was significantly higher in the PCOS group (30.43 ± 5.02 mm vs. 28.47 ± 5.21 mm, *p* < 0.001).

Fasting glucose, alanine aminotransferase (ALT), aspartate aminotransferase (AST), creatinine, and cholesterol levels were similar across the groups. However, fasting insulin (14.26 ± 2.44 μU/mL vs. 12.41 ± 2.38 μU/mL, *p* = 0.009) and HOMA-IR (3.29 ± 0.55 vs. 2.87 ± 0.49, *p* < 0.001) were found to be significantly higher in the PCOS group.

The PCOS group had significantly higher levels of LH, the LH/FSH ratio, total testosterone, free testosterone, androstenedione, and DHEAS (*p* < 0.001). The control group had significantly higher levels of FSH (*p* = 0.003) and SHBG (*p* < 0.001). There was no significant difference between the groups for estradiol, prolactin, SBP, DBP, or heart rate. The demographic, anthropometric, laboratory, and cardiovascular measurement results of the study group are presented in Table 1.

**Figure 1:**
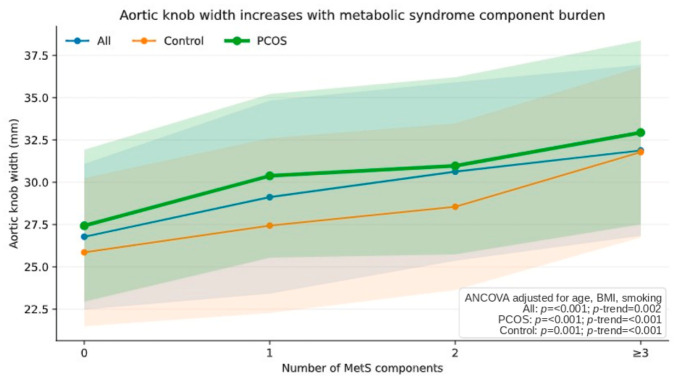
Trends of aortic knob width according to metabolic syndrome burden. The points represent the adjusted mean values of aortic knob width obtained from ANCOVA models that were adjusted for age, BMI, and smoking status. The connecting lines show the direction and size of the change in aortic knob width as the number of metabolic syndrome components increases within each group. The shaded areas surrounding each line represent the 95% confidence intervals around the adjusted mean values derived from the ANCOVA models. *p* values indicate overall group differences, while p-trend values reflect the significance of a linear trend across metabolic syndrome component categories. PCOS, polycystic ovary syndrome; ANCOVA, analysis of covariance; BMI, body mass index.

Participants were classified into four subgroups based on the number of MetS components they had: ‘0, 1, 2, and ≥3’. According to the analysis results, the prevalence of MetS was 24.25% among all participants, 39% among PCOS patients, and 9.5% in the control group. Visualized trend analysis shows that AKW increases gradually with the burden of MetS, and this increase is more pronounced in the PCOS group (Figure 1). The progressively increasing slope observed in the PCOS group highlights a stronger association between metabolic syndrome burden and AKW compared to the control group.

To further explore the observed difference in MetS prevalence between groups, the prevalence of each individual MetS component was analyzed separately (Supplementary Table S1). Women with PCOS exhibited significantly higher frequencies of elevated waist circumference (41.0% vs. 24.0%, *p* < 0.001), elevated triglycerides (27.0% vs. 16.0%, *p* = 0.007), reduced HDL cholesterol (35.5% vs. 21.5%, *p* = 0.002), elevated blood pressure (24.5% vs. 10.5%, *p* < 0.001), and elevated fasting glucose (19.0% vs. 9.0%, *p* = 0.004) compared with controls. Consequently, the prevalence of MetS was substantially higher in the PCOS group than in the control group (39.0% vs. 9.5%, *p* < 0.001).

**Figure 2:**
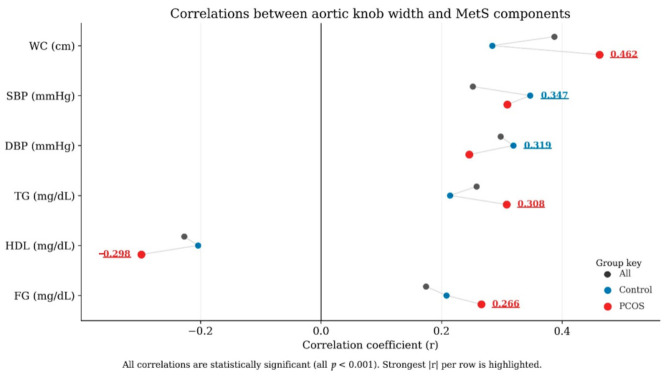
Forest plot of correlation coefficients (r) between aortic knob width and MetS components in the overall cohort, PCOS patients, and controls. Points represent r values; the vertical line indicates no correlation (r = 0). Positive correlations appear to the right and negative correlations to the left. MetS, metabolic syndrome; WC, waist circumference; SBP, systolic blood pressure; DBP, diastolic blood pressure; TG, triglyceride; HDL, high-density lipoprotein; FG, fasting glucose; PCOS, polycystic ovary syndrome.

Correlation analysis revealed that AKW was significantly associated with all components of MetS (*p* < 0.001 for all). Positive correlations were observed with WC, blood pressure, TG, and fasting glucose, while a negative correlation was observed with HDL cholesterol. Figure 2 shows that correlation coefficients were higher in the PCOS group than in the control group. The corresponding correlation coefficients, 95% CIs, and significance levels for each subgroup are presented in Supplementary Table S2.

Stratification of PCOS patients according to AKW quartiles revealed a clear and progressive deterioration in individual MetS components. WC, systolic and diastolic blood pressure, TG levels, and fasting glucose showed progressively higher adjusted mean values in higher AKW quartiles, while HDL cholesterol showed a gradual decline.

**Figure 3:**
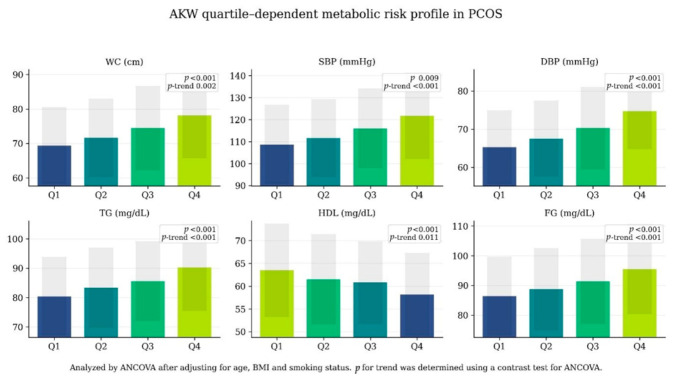
Visual summary of metabolic syndrome component trends across AKW quartiles in PCOS patients. Bar heights represent adjusted mean values across AKW quartiles (Q1–Q4). Color gradient emphasizes the stepwise change across quartiles (progressively darker tones denote higher quartiles; reversed gradient for HDL due to decreasing trend). Semi-transparent bands indicate variability around the mean (±standard deviation). Panel annotations report the overall *p* value and *p* for trend. AKW, aortic knob width; PCOS, polycystic ovary syndrome; WC, waist circumference; SBP, systolic blood pressure; DBP, diastolic blood pressure; TG, triglyceride; HDL, high-density lipoprotein; FG, fasting glucose.

These associations remained significant after adjusting for BMI, age, and smoking status. All components exhibited significant linear trends across AKW quartiles (*p* < 0.05 for all trends). The strongest trends were observed in blood pressure and fasting glucose levels (Figure 3).

**Figure 4:**
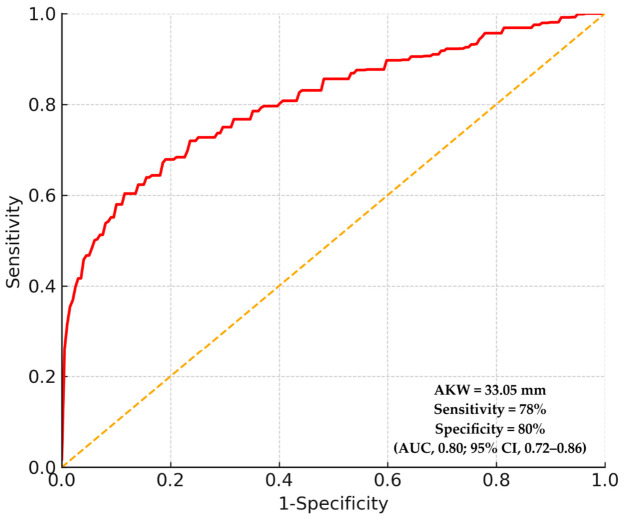
ROC curve illustrating the association between AKW and the presence of MetS within the study cohort of women with PCOS. AKW, aortic knob width; AUC, area under the curve; CI, confidence interval; ROC, receiver operating characteristic; MetS, metabolic syndrome; PCOS, polycystic ovary syndrome.

ROC curve analysis demonstrated that AKW had significant discriminatory ability for identifying MetS within the study cohort, showing moderate-to-good performance. ROC analysis identified an exploratory AKW threshold of 33.05 mm, which yielded a sensitivity of 78% and a specificity of 80% for discriminating participants with and without MetS [area under the curve (AUC): 0.80; 95% CI: 0.72–0.86; *p* < 0.001] (Figure 4).

## 4. Discussion

In our study investigating the relationship between AKW and MetS in patients with PCOS, we found that patients with elevated AKW were associated with the presence of MetS within the study cohort. Our findings revealed higher AKW values in women with PCOS than in a matched control group. There was also a marked, stepwise increase in AKW associated with an increased MetS burden. This association was notably more pronounced in the PCOS group, suggesting a stronger link between vascular remodeling and metabolic dysfunction in this population. We found that AKW demonstrated moderate-to-good discriminatory performance for identifying MetS in women with PCOS.

PCOS is a complex condition that affects the reproductive and metabolic systems. PCOS is characterized by chronic anovulation and hyperandrogenism and is also associated with numerous metabolic and endocrine disorders [4,5]. Women with PCOS often first seek medical care due to menstrual irregularities, infertility, and hirsutism. However, insulin resistance, obesity, dyslipidemia, hypertension, and MetS are also prevalent among this population. These conditions place women with PCOS at a higher long-term risk of developing type 2 DM and CVD [7,32]. Extensive population-based studies and meta-analyses consistently demonstrate that women with PCOS have an increased risk of CVD, independent of traditional risk factors [33,34].

Although there remains ongoing debate over whether women with PCOS have a higher risk of CVD independent of known CVD risk factors, early-stage manifestations of PCOS, such as menstrual irregularities, suggest that these women may have a higher CVD risk factor profile. Therefore, screening for CVD risk factors and taking preventive measures should be encouraged to prevent long-term cardiometabolic outcomes [7,35]. Consistent with these pathophysiological assessments, we observed significant correlations between AKW and all components of MetS, including WC, blood pressure, TG, and fasting glucose. Conversely, we found an inverse relationship with HDL cholesterol. Notably, these correlations were stronger in the PCOS group, which further supports the hypothesis that PCOS is characterized by increased cardiometabolic interactions.

Compared to the control group, women with PCOS have higher levels of LDL cholesterol and TG, as well as lower levels of HDL cholesterol [36]. A significant proportion of women with PCOS have obesity and insulin resistance [6]. PCOS is characterized by impaired glucose tolerance, insulin resistance, and hyperinsulinemia, which result from molecular abnormalities in insulin activity [37]. Adipose tissue dysfunction has been identified as a contributing factor to the insulin resistance observed in PCOS [6]. It has been shown that 95% of obese women with PCOS and 75% of lean women with PCOS have insulin resistance [38,39]. Additionally, subclinical inflammation is common in this condition and has been found to be associated with insulin resistance [7,8]. PCOS is also associated with hypertension [40]. Although this risk appears to be independent of BMI, obesity further increases it [41]. A meta-analysis by Lim et al. showed that women with PCOS have significantly higher rates of being overweight or obese, as well as central obesity, compared to women without PCOS [42]. Consistent with these findings, our results showed that women with PCOS had statistically significantly higher insulin levels, HOMA-IR values, and WC compared to the matched control group. Additionally, the PCOS group had a significantly higher prevalence of MetS, which further supports the close interaction between adiposity, insulin resistance, and cardiometabolic risk in this population.

MetS is a serious condition characterized by the presence of multiple risk factors, including insulin resistance, hyperglycemia, dyslipidemia, central obesity, and hypertension [43]. Numerous studies have shown that MetS is associated with an increased risk of developing type 2 DM and CVD [14,15]. A 2010 meta-analysis found that the presence of MetS was correlated with a twofold elevation in the risk of CVD, CVD-related mortality, and stroke, alongside a 1.5-fold increase in all-cause mortality. Furthermore, the analysis identified an approximately twofold rise in the risk of myocardial infarction [15]. Insulin resistance results in hyperinsulinemia and hyperglycemia, conditions that subsequently induce peripheral vasoconstriction and sodium retention. Production of very-low-density lipoproteins (VLDL) in the liver also increases, resulting in low HDL cholesterol, high apolipoprotein B, high LDL cholesterol, and hypertriglyceridemia. Consequently, atherosclerosis ensues. Due to these lipid imbalances, individuals with MetS exhibit a prothrombotic and proinflammatory state [13,44,45]. Adipocytes secrete substances such as leptin, tumour necrosis factor-alpha (TNF-α), resistin, and adiponectin, which lead to insulin resistance. In other words, central obesity causes systemic hypertension and dyslipidemia both by triggering insulin resistance and independently [46,47,48]. Consistent with these pathophysiological mechanisms, our findings revealed that AKW increased gradually with the burden of MetS components and showed a significant association with all individual MetS parameters. WC, blood pressure, TG, and fasting glucose showed a positive correlation with AKW, whereas HDL cholesterol exhibited an inverse correlation.

In recent years, the metabolic aspect of PCOS has received increasing attention. Most women with PCOS have been found to have metabolic disorders, insulin resistance, and hyperinsulinemia [49]. Studies have reported that MetS is more common in women with PCOS due to the higher prevalence of obesity, visceral obesity and insulin resistance in this population [38,50]. Patients with PCOS have been shown to have a higher risk of developing MetS compared to BMI-matched healthy controls, a finding that has also been confirmed in non-obese women with PCOS [16,17]. Consistent with these observations, our study demonstrated that the prevalence of MetS was significantly higher in women with PCOS compared to an age- and BMI-matched control group. Insulin levels and HOMA-IR values were significantly elevated in the PCOS group, indicating pronounced insulin resistance. Additionally, the observed increase in AKW in these patients suggests that this adverse metabolic profile may already be associated with early-stage structural vascular changes. Because women with previously diagnosed DM or hypertension were excluded, the study population primarily represented individuals without established cardiometabolic disease. Although MetS could still be diagnosed based on abnormal metabolic measurements, the exclusion of participants with known DM or hypertension may have reduced the representation of more severe metabolic phenotypes and may limit the generalizability of the findings to women with advanced cardiometabolic disease.

Radiography is one of the most cost-effective and readily available imaging methods in a clinical setting and can provide important insights into the cardiovascular system. The AKW is a simple measurement representing the radiographic projection of the aortic arch and the descending aorta [19]. It has been shown that an increase in AKW is associated with various cardiovascular conditions, such as hypertension, atherosclerosis, and cardiac dysfunction [21,23]. Furthermore, AKW, which reflects a combination of aortic stiffening, dilation, and wall thickening, is considered not only a marker of local changes but also an indicator of systemic atherosclerotic burden [25].

In this context, our finding that AKW is closely associated with both the presence of MetS and metabolic burden suggests that AKW may serve as a comprehensive reflection of cardiometabolic risk.

While previous studies have demonstrated an association between AKW and MetS [20], our findings extend these observations by showing that this association remains evident in women with PCOS, a population at increased cardiometabolic risk. In addition, ROC analysis indicated that AKW exhibited moderate-to-good discriminatory ability for distinguishing participants with and without MetS within the study cohort. The discriminatory ability of AKW observed in the present study was evaluated exclusively within the study cohort, and no external validation was performed. Therefore, these findings should be considered exploratory and require confirmation in independent prospective cohorts before any broader clinical application can be considered.

Although our analyses were adjusted for age, BMI, and smoking status, residual confounding cannot be completely excluded. Variables such as physical activity, dietary habits, socioeconomic status, PCOS duration, family history of CVD, and inflammatory biomarkers were not consistently available in this retrospective dataset and therefore could not be included in the analyses. Furthermore, insulin resistance, although available, was not incorporated into the adjustment model because it may represent an intermediate biological mechanism linking PCOS-related metabolic dysfunction and vascular remodeling. Consequently, the observed associations between AKW and MetS should be interpreted as associations within the study cohort rather than evidence of an independent causal relationship.

Current guidelines recommend advanced imaging techniques for the assessment of aortic diameter [51]. However, considering the limitations of these methods regarding cost, accessibility, and radiation exposure, AKW measurement via routine chest radiograph may offer a highly accessible and cost-effective alternative in clinical practice. Therefore, it is suggested that AKW could serve as a supplementary parameter for the early identification of cardiometabolic risk, particularly in resource-limited settings.

### 4.1. Clinical Implications

AKW may be investigated as a potential adjunctive radiographic marker associated with cardiometabolic risk in women with PCOS. Given its simplicity and wide availability, AKW could represent a promising parameter for future risk stratification strategies. The ROC-derived threshold of 33.05 mm should be considered an exploratory finding generated from the current study population. Because this value has not been externally validated, it should not be regarded as a clinically established cutoff and requires confirmation in independent prospective cohorts. However, because this threshold was derived and evaluated within the same cohort, it should be regarded as exploratory and hypothesis-generating rather than clinically actionable. External validation studies are required before its potential clinical applicability can be assessed.

### 4.2. Strengths of the Study

This study has several strengths, including the use of an age- and BMI-matched control group, a comprehensive assessment of metabolic and hormonal parameters, and the evaluation of AKW using a reproducible and widely accessible imaging modality. Furthermore, the consistent associations observed between AKW and MetS-related parameters, together with its moderate-to-good discriminatory performance for identifying MetS, enhance the overall robustness and clinical relevance of the findings.

### 4.3. Limitations

This study has several limitations. First, its retrospective observational design precludes causal inference and limits the ability to determine the temporal relationship between AKW and MetS. Therefore, the observed associations should be interpreted as hypothesis-generating rather than evidence of a causal relationship.

Second, although analyses were adjusted for age, BMI, and smoking status, residual confounding cannot be completely excluded. Several potentially relevant variables, including physical activity, dietary habits, socioeconomic characteristics, family history of CVD, duration of PCOS, and inflammatory biomarkers, were not consistently available in the retrospective records and therefore could not be incorporated into the analyses. In addition, differences in smoking exposure and intensity could not be fully assessed despite adjustment for smoking status.

Third, a chest radiograph obtained within the specified time frame is necessary. As chest radiography is not routinely conducted for all women with PCOS, only those participants with available posteroanterior chest radiographs were included. Consequently, selection bias cannot be excluded, and the study population might not fully represent the broader PCOS population. Therefore, the generalizability of our findings should be approached with caution.

Fourth, women with previously diagnosed DM, hypertension, and other major chronic diseases were excluded to minimize confounding. Although MetS could still be identified based on abnormal metabolic measurements, this approach may have reduced the representation of more severe cardiometabolic phenotypes and may limit the generalizability of the findings to women with advanced cardiometabolic disease.

Fifth, ultrasonographic evaluations used for the diagnosis of PCOS could not be standardized retrospectively, and interobserver and intraobserver reproducibility could not be assessed as was performed for AKW measurements. Furthermore, ultrasound technology and recommendations for defining polycystic ovary morphology have evolved over time. Because this study was retrospective, diagnostic classifications were based on the criteria routinely applied during the study period, and no retrospective reclassification was performed. Therefore, some differences may exist compared with more recent ultrasound-based definitions of polycystic ovary morphology.

Finally, the ROC-derived threshold and discriminatory performance of AKW were evaluated exclusively within the present cohort, and no external validation was performed. Therefore, these findings should be considered exploratory and require confirmation in independent prospective multicenter studies before broader clinical application can be considered.

## 5. Conclusions

In conclusion, AKW was significantly associated with the presence and burden of metabolic syndrome among women with PCOS in this selected study cohort. Higher AKW values were associated with less favorable cardiometabolic profiles and a greater number of MetS components. However, owing to the retrospective design, the possibility of residual confounding, and the absence of external validation, these findings should be interpreted as associative rather than predictive or causal. AKW may therefore be considered a potential adjunctive radiographic marker associated with cardiometabolic risk in women with PCOS. However, prospective multicenter studies with comprehensive adjustment for potential confounding factors and external validation are required before routine clinical implementation or adoption of a specific cutoff value can be recommended.

## Figures and Tables

**Table 1:** Data on demographic, anthropometric, laboratory, and cardiovascular parameters of the study groups.

Variable	All (*n* = 400)	PCOS (*n* = 200)	Control (*n* = 200)	*p* Value
Age, years	24.18 ± 4.16	23.92 ± 4.17	24.48 ± 4.08	0.306
BMI, kg/m^2^	27.06 ± 4.15	27.18 ± 4.82	26.92 ± 4.37	0.266
WC, cm	72.68 ± 11.94	73.39 ± 11.77	71.98 ± 12.08	0.001
HC, cm	93.54 ± 15.56	93.28 ± 15.44	93.76 ± 15.82	0.502
Aortic knob width, mm	29.64 ± 5.09	30.43 ± 5.02	28.47 ± 5.21	<0.001
Smoking, n (%)	54 (13.5%)	22 (11%)	32 (16%)	0.108
Fasting glucose, mg/dL	91.38 ± 14.46	90.49 ± 14.12	92.88 ± 13.98	0.306
Insulin, μU/mL	13.22 ± 2.36	14.26 ± 2.44	12.41 ± 2.38	0.009
HOMA-IR	3.09 ± 0.52	3.29 ± 0.55	2.87 ± 0.49	<0.001
ALT, U/L	28.08 ± 4.75	27.12 ± 4.63	28.46 ± 5.25	0.203
AST, U/L	27.63 ± 4.82	26.84 ± 4.98	28.22 ± 4.88	0.381
Creatinine, mg/dL	0.82 (0.68–1.02)	0.82 (0.68–1.01)	0.80 (0.67–1.03)	0.408
Triglycerides, mg/dL	85.09 ± 14.38	84.91 ± 13.86	85.12 ± 14.22	0.392
Total cholesterol, mg/dL	189.45 ± 31.05	190.86 ± 30.87	188.74 ± 30.44	0.273
HDL cholesterol, mg/dL	61.24 ± 9.78	60.99 ± 9.57	61.62 ± 9.61	0.581
LDL cholesterol, mg/dL	101.21 ± 16.86	103.12 ± 17.08	100.24 ± 16.82	0.175
FSH, mIU/mL	5.58 ± 1.07	4.32 ± 1.05	6.84 ± 1.05	0.003
LH, mIU/mL	7.56 ± 1.22	9.16 ± 1.36	5.98 ± 1.01	<0.001
LH/FSH ratio	1.51 ± 0.25	2.13 ± 0.34	0.85 ± 0.13	<0.001
Estradiol, pg/mL	63.78 ± 11.06	62.24 ± 10.26	65.57 ± 11.37	0.089
Total testosterone, ng/dL	64.55 ± 12.84	89.24 ± 14.16	41.67 ± 6.78	<0.001
Free testosterone, ng/dL	2.09 ± 0.38	2.86 ± 0.46	1.24 ± 0.21	<0.001
Androstenedione, ng/mL	3.18 ± 0.84	4.36 ± 0.72	2.12 ± 0.36	<0.001
DHEAS, mg/dL	272.34 ± 44.38	296.58 ± 47.07	252.16 ± 40.67	<0.001
SHBG, nmol/L	54.8 (34.3–76.7)	45.2 (32.3–62.4)	64.3 (36.4–89.6)	<0.001
Prolactin, ng/mL	13.88 ± 2.68	14.38 ± 2.36	13.26 ± 2.41	0.612
SBP, mmHg	113.37 ± 18.17	114.47 ± 18.35	112.19 ± 17.96	0.447
DBP, mmHg	69.82 ± 10.21	69.45 ± 10.07	70.28 ± 10.37	0.128
Heart rate, bpm	68.58 ± 11.23	68.12 ± 11.05	69.08 ± 10.98	0.512

Values are presented as mean ± standard deviation and median (25–75%). *p* values were derived from independent-samples *t*-tests, Mann–Whitney U tests, or chi-square tests, as appropriate according to variable distribution and measurement scale. Because of variable-specific missing data, the number of observations differed across variables. Therefore, values presented in the ‘All’ column may not exactly correspond to the arithmetic average of the PCOS and control groups. PCOS, polycystic ovary syndrome; BMI, body mass index; WC, waist circumference; HC, hip circumference; HOMA-IR, homeostasis model assessment of insulin resistance; ALT, alanine aminotransferase; AST, aspartate aminotransferase; HDL, high-density lipoprotein; LDL, low-density lipoprotein; FSH, follicle-stimulating hormone; LH, luteinizing hormone; DHEAS, dehydroepiandrosterone-sulfate; SHBG, sex hormone-binding globulin; SBP, systolic blood pressure; DBP, diastolic blood pressure.

## Data Availability

Data are available on request due to privacy.

## Supplementary Materials

The following supporting information can be downloaded at: https://www.mdpi.com/article/10.3390/jcm15135273/s1, Table S1. Prevalence of individual metabolic syndrome components and overall metabolic syndrome in women with PCOS and controls. Table S2. Correlations between aortic knob width and metabolic syndrome components with corresponding 95% confidence intervals.

## Abbreviations

The following abbreviations are used in this manuscript:

PCOS	Polycystic ovary syndrome
MetS	Metabolic syndrome
AKW	Aortic knob width
ROC	Receiver operating characteristic
DM	Diabetes mellitus
CVD	Cardiovascular disease
BMI	Body mass index
WC	Waist circumference
HDL	High-density lipoprotein
TG	Triglyceride
CIMT	Carotid intima-media thickness
β-hCG	Beta-human chorionic gonadotropin
HC	Hip circumference
SBP	Systolic blood pressure
DBP	Diastolic blood pressure
LDL	Low-density lipoprotein
HOMA-IR	Homeostasis model assessment of insulin resistance
FSH	Follicle-stimulating hormone
LH	Luteinizing hormone
DHEAS	Dehydroepiandrosterone sulfate
SHBG	Sex hormone-binding globulin
E2	Estradiol
ICC	Intraclass correlation coefficient
CI	Confidence interval
SD	Standard deviation
ANCOVA	Analysis of covariance
ALT	Alanine aminotransferase
AST	Aspartate aminotransferase
FG	Fasting glucose
AUC	Area under the curve
VLDL	Very-low-density lipoprotein
TNF-α	Tumour necrosis factor-alpha
