# Supplementary material for: The Relationship Between Aortic Knob Width and Metabolic Syndrome in Women with Polycystic Ovary Syndrome
Source: J Clin Med. 2026 Jul 6;15(13):5273. doi: 10.3390/jcm15135273 (PMC13362919; doi:10.3390/jcm15135273)
Supplement: Supplementary file 1 [file jcm-15-05273-s001.zip › jcm-4352579-supplementary.pdf]

Supplementary Tables

Table S1. Prevalence of individual metabolic syndrome components and overall metabolic syndrome in women with PCOS and controls.

MetS Component	PCOS (*n* = 200)	Control (*n* = 200)	*p* Value
Elevated waist circumference, n (%)	82 (41.0%)	48 (24.0%)	<0.001
Elevated triglycerides, n (%)	54 (27.0%)	32 (16.0%)	0.007
Reduced HDL cholesterol, n (%)	71 (35.5%)	43 (21.5%)	0.002
Elevated blood pressure, n (%)	49 (24.5%)	21 (10.5%)	<0.001
Elevated fasting glucose, n (%)	38 (19.0%)	18 (9.0%)	0.004
MetS (≥3 components), n (%)	78 (39.0%)	19 (9.5%)	<0.001

MetS, metabolic syndrome; PCOS, polycystic ovary syndrome; HDL, high-density lipoprotein.

Table S2. Correlations between aortic knob width and metabolic syndrome components with corresponding 95% confidence intervals.

Variable	Group	r	95% CI	*p* Value
WC	All	0.387	0.30–0.47	<0.001
WC	PCOS	0.462	0.35–0.56	<0.001
WC	Control	0.284	0.15–0.41	<0.001
SBP	All	0.252	0.16–0.34	<0.001
SBP	PCOS	0.309	0.18–0.43	<0.001
SBP	Control	0.347	0.22–0.46	<0.001
DBP	All	0.298	0.21–0.39	<0.001
DBP	PCOS	0.246	0.11–0.37	<0.001
DBP	Control	0.319	0.19–0.44	<0.001
TG	All	0.258	0.16–0.35	<0.001
TG	PCOS	0.308	0.18–0.43	<0.001
TG	Control	0.214	0.08–0.34	<0.001
HDL	All	−0.227	−0.32 to −0.13	<0.001
HDL	PCOS	−0.298	−0.42 to −0.17	<0.001
HDL	Control	−0.204	−0.33 to −0.07	<0.001
FG	All	0.174	0.08–0.27	<0.001
FG	PCOS	0.266	0.13–0.39	<0.001

Variable	Group	r	95% CI	*p* Value
FG	Control	0.208	0.07–0.34	<0.001

CI, confidence interval; WC, waist circumference; PCOS, polycystic ovary syndrome; SBP, systolic blood pressure; DBP, diastolic blood pressure; TG, triglyceride; HDL, high-density lipoprotein; FG, fasting glucose.
